# The key role of heterochromatin in the phenotypic manifestation of the In(1)sc8 inversion disrupting the achaete-scute complex in Drosophila melanogaster

**DOI:** 10.18699/vjgb-25-44

**Published:** 2025-06

**Authors:** T.D. Kolesnikova, M.N. Balantaeva, G.V. Pokholkova, O.V. Antonenko, I.F. Zhimulev

**Affiliations:** Institute of Molecular and Cellular Biology of the Siberian Branch of the Russian Academy of Sciences, Novosibirsk, Russia Novosibirsk State University, Novosibirsk, Russia; Novosibirsk State University, Novosibirsk, Russia; Institute of Molecular and Cellular Biology of the Siberian Branch of the Russian Academy of Sciences, Novosibirsk, Russia; Institute of Molecular and Cellular Biology of the Siberian Branch of the Russian Academy of Sciences, Novosibirsk, Russia; Institute of Molecular and Cellular Biology of the Siberian Branch of the Russian Academy of Sciences, Novosibirsk, Russia

**Keywords:** achaete-scute complex, AS-C, position effect, position effect modifiers, heterochromatin, inversions, Drosophila melanogaster, Rif1, Su(var)3-9, achaete-scute complex, AS-C, эффект положения, модификаторы эффекта положения, гетерохроматин, инверсии, Drosophila melanogaster, Rif1, Su(var)3-9

## Abstract

The achaete-scute complex (AS-C) is a locus approximately 90 kbp in length, containing multiple enhancers. The local expression of the achaete and scute genes in proneural clusters of Drosophila melanogaster imaginal discs results in the formation of a well-defined pattern of macrochaetae in adult flies. A wide variety of easily analyzed phenotypes, along with the direct connection between individual regulatory elements and the development of specific setae make this locus a classic model in developmental genetics. One classic AS-C allele is sc8, which arose as a result of the In(1) sc8 inversion. One breakpoint of this inversion lies between the ac and sc genes, while the second is in the pericentromeric heterochromatin of chromosome X, within satellite block 1.688. The heterochromatic position of the breakpoint raised the question of whether position effect variegation contributes to the disruption of normal locus function in the In(1)sc8 flies. However, conflicting results were obtained. Previously, we found that a secondary inversion, In(1)19EHet, arose spontaneously in one of the stocks of the In(1)sc8 BDSC line, transferring most of the heterochromatin from the ac gene to the 19E region of the X chromosome. Here, we demonstrate that the In(1)19EHet inversion leads to complete rescue of the number of posterior supraalar (PSA) and partial rescue of the number of dorsocentral (DC) macrochaetes observed in the original In(1)sc8 line. The same rescue of the macrochaetes pattern was observed when the In(1)sc8 inversion was introduced into a strain with the Su(var)3-906 position effect modifier. Combining the inversion with the Rif11 mutation, a conserved factor determining late replication and underreplication, does not restore the normal pattern of bristles. Our data indicate that the phenotype of flies carrying the In(1) sc8 inversion, associated with a disturbance in bristle development, is determined by the effect of heterochromatin on the distal part of the locus. This model can be used to test the influence of various factors on the position effect variegation caused by heterochromatin. Another phenotypic manifestation of In(1)sc8, a decreased proportion of males in the offspring, was independent of the proximity of the distal part of AS-C to heterochromatin and was not affected by the Rif11 mutation.

## Abstract (ru)

Локус achaete-scute (achaete-scute complex, AS-C) занимает около 90 т. п. н. и содержит множественные энхансеры. Локальная экспрессия генов achaete и scute в пронейральных кластерах имагинальных дисков Drosophila melanogaster приводит к формированию детерминированного рисунка макрохет у взрослых мух. Большое многообразие легко анализируемых видимых фенотипов, прямая связь между отдельными регуляторными элементами и развитием конкретных щетинок сделали этот локус классическим модельным объектом генетики развития. Одним из самых известных AS-C является аллель sc8, возникший в результате инверсии In(1)sc8. Дистальная точка разрыва этой инверсии лежит между генами ac и sc, проксимальная – в прицентромерном гетерохроматине хромосомы Х, в блоке сателлита 1.688. Гетерохроматиновое положение точки разрыва поднимало вопрос о роли эффекта положения мозаичного типа в нарушении нормальной работы локуса у носителей инверсии, но были получены противоречивые результаты. Ранее мы обнаружили, что в одном из стоков линии, несущей In(1)sc8, спонтанно возникла вторичная инверсия In(1)19EHet, которая переносит большую часть гетерохроматина от гена ac в локус, соответствующий району 19E политенной Х-хромосомы. В настоящей статье мы показали, что инверсия In(1)19EHet приводит к полному восстановлению числа задних супраалярных и частичному – дорзо-центральных щетинок, наблюдаемых у мух исходной линии In(1) sc8. Точно такое же восстановление паттерна щетинок мы увидели при введении в линию с инверсией In(1) sc8 модификатора эффекта положения – мутации Su(var)3-906. Введение же в линию с инверсией мутации по гену Rif1 – консервативного фактора, определяющего позднюю репликацию и недорепдикацию ДНК в клетках D. melanogaster, не приводит к восстановлению нормального паттерна щетинок. Наши данные указывают на то, что фенотип мух – носителей инверсии In(1)sc8, связанный с нарушением развития щетинок, определяется эффектом гетерохроматина на дистальную часть локуса и может использоваться для проверки влияния различных факторов на вызываемый гетерохроматином эффект положения гена. Еще одно фенотипическое проявление In(1) sc8 – снижение доли самцов в потомстве – оказалось независимым от соседства дистальной части AS-C с гетерохроматином. Этот фенотип также не восстанавливался на фоне мутации в гене Rif1.

## Introduction

**Fig. 1.**
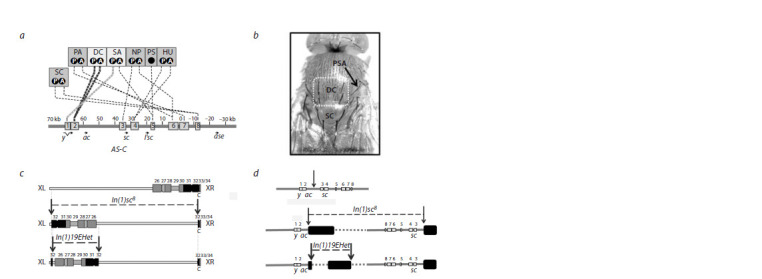
The double inversion In(1)sc8+19EHet splits the AS-C complex into two parts, attaching each part to a block of pericentric heterochromatin, resulting in altered spatial arrangement of the locus genes and their regulators, as well as a potential position effect variegation. The double inversion In(1)sc8+19EHet splits the AS-C complex into two parts, attaching each part to a block of pericentric heterochromatin, resulting in altered spatial arrangement of the locus genes and their regulators, as well as a potential position effect variegation.

External mechanoreceptors in Drosophila are represented by bristles of varying sizes – macro- and microchaetae. Macrochaetae form a stable structural composition known as the bristle pattern, in which each macrochaeta occupies a strictly defined position. The formation of the bristle pattern begins with the establishment of its precursor in the imaginal disc. The specificity of the future mechanoreceptor positions is determined by the local expression of two proneural genes – achaete (ac) and scute (sc) – that are part of the AS-C complex (Modolell, Campuzano, 1998; Gómez-Skarmeta et al., 2003; Bukharina, Furman, 2015; Troost et al., 2015; Furman, Bukharina, 2019). The AS-C occupies approximately 90 kilobase pairs of DNA and consists of four genes (achaete, scute, lethal of scute, and asense) that encode transcription factors involved in the regulation of nervous system development. Multiple enhancers have been identified and characterized within the locus, each of which determines the function of the complex genes in specific proneural clusters, giving rise to the corresponding macrochaeta (Fig. 1a, b). Under normal conditions, the ac and sc genes are regulated by the same enhancers, and their products are produced in the same cells. Furthermore, the functions of these genes are partially redundant (Modolell, Campuzano, 1998).

The inversion In(1)sc8 (Fig. 1c, d) splits the AS-C locus apart between the ac and sc genes and connects both parts to 1.688 satellite blocks in pericentric heterochromatin (Miller et al., 2016). In this case, the distal enhancers remain with the portion of the complex carrying ac, while the others are translocated by the inversion along with the sc gene (Fig. 1d). It has been demonstrated that in this scenario, one part of the proneural clusters expresses only ac, while the complementary part expresses only sc, and their effects complement each other (Gómez-Skarmeta et al., 1995). This likely explains the weak phenotype observed in the inversion-bearing flies, as phenotypic changes involve only a few groups of macrochaetae. In inversion carriers, a reduction in the number of supra-alars (SA) may occur, and additional bristles can be found on the scutellum and in the dorso-central region (García-Bellido, 1979; Lindsley, Zimm, 1992).

The inversion In(1)sc8 was obtained in the laboratory of A.S. Serebrovsky through irradiation of flies with the w a genotype (Sidorov, 1931). The line In(1)sc8, sc8 y 31d w a was transferred to the Bloomington Drosophila Stock Center (BDSC) in 1986 and assigned the number #798. In 2012, this line was split into two independent sublines (#798 main copy and #798 backup copy). In 2020, after replacing the second chromosome in the #798 main copy line with a chromosome carrying the Rif11 mutation, which completely suppresses the underreplication of heterochromatic sequences in polytene chromosomes, an additional inversion was discovered. This finding occurred during the analysis of polytene chromosome preparations. The breakpoints of the new inversion were characterized cytologically and molecularly (Kolesnikova et al., 2022). The new inversion was named “In(1)19EHet”, and the complex chromosomal rearrangement involving both inversions was designated “In(1)sc8+19EHet”. Schematic diagrams illustrating the positions of the breakpoints are shown in Figure 1 (c, d).

Numerous genetic and environmental factors are involved in bristle development. The phenotype depends on the genetic background, developmental temperature of the flies, and their sex (Child, 1935; Furman, Ratner, 1977). The existence of two lines with a common origin, similar genetic backgrounds (both lines are descendants of flies that were split from a single tube in 2012), and differing by the In(1)19EHet inversion, which removes a large portion of heterochromatin from one of the breakpoints of the In(1)sc8 inversion, provides a unique opportunity to investigate the influence of heterochromatin on the phenotype of inversion carriers.

## Materials and methods

Flies were cultured at temperatures of 18 or 25 ºC, avoiding overcrowding, on a standard medium composed of: agaragar – 10 g, pressed yeast – 100 g, cornmeal – 50 g, sugar – 20 g, and raisins – 40 g per 1 liter of water. The Table lists the fly lines used in the study and the sample sizes.

**Table 1.**
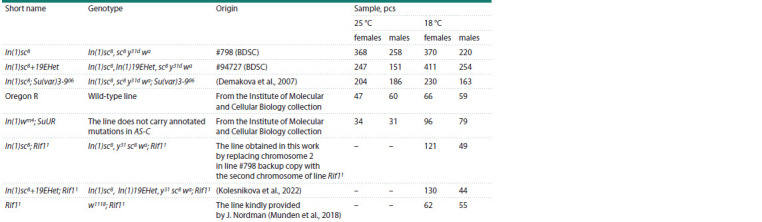
The D. melanogaster lines used in the study and the sizes of the corresponding samples

Flies were examined under a binocular loupe. For each fly, information was recorded in the table regarding the number of posterior supraalar bristles (2 (normal), 1, or 0), and the presence/ absence of abnormalities in the number of dorsocentral and scutellar bristles (normal, extra/missing bristles).

Statistical analysis was conducted using Excel, and the Chi-square test was employed to assess the significance of the differences ( p-value).

## Results

Based on literature data regarding the phenotype of In(1)sc8, we selected three groups of macrochaetae for detailed analysis: posterior supra-alar (PSA), dorsocentral (DC), and scutellar (SC). We analyzed the number of these bristles on the notum of flies from the lines In(1)sc8, y 31 sc8 w a (hereinafter referred to for simplicity as In(1)sc8), and In(1)sc8 In(1)19EHet, sc8 y 31d w a (hereinafter In(1)sc8+19EHet). As control lines, for which mutations disrupting macrochaeta development have not been described, we used the wild-type line Oregon R and several lines from the laboratory of molecular cytogenetics, IMCB SB RAS.

Additionally, we included the line In(1)sc8; Su(var)3-906 in our analysis, where the In(1)sc8 inversion occurs against a strong mosaic position effect modifier. In the control lines Oregon R and In(1)w m4; SuUR, we did not observe any abnormalities in the number of PSA bristles (n = 236 and 240, respectively). The proportion of flies with additional DC bristles did not exceed 2 % in the Oregon R line and 4 % in the In(1)w m4; SuUR. Additionally, in the Oregon R line, the proportion of flies with abnormalities in the number of scutellar bristles reached 6 %, while in the In(1)w m4; SuUR line, it did not exceed 1 %.

**Fig. 2.**
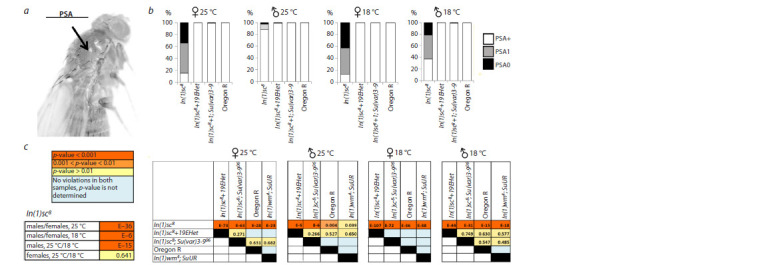
The effect of heterochromatin removing and the position effect variegation modifier Su(var)3-906 on the development of posterior supra-alar bristles in In(1)sc8 flies a – a typical phenotype of the In(1)sc8 line showing the absence of the posterior supraalar bristle (arrow indicates the position where the PSA is normally located); b – the proportion of flies with abnormalities in the number of posterior supraalar bristles (PSA+ – normal phenotype, PSA1 – presence of one bristle, PSA0 – absence of bristles) in the lines In(1)sc8, In(1)sc8+19EHet, In(1)sc8; Su(var)3-906, and two control lines Oregon R and In(1)wm4; SuUR. Results for males and females are provided separately, as well as for flies reared at different temperatures; c – pairwise comparison of the proportions of flies with altered bristle counts among different genotypes. The p-value for each comparison, calculated using the Chi-square test, is indicated

In flies of the In(1)sc8 line, it is characteristic to observe the absence of one or both PSA bristles (Fig. 2a). Only 14–15 % of females reared at both 25 and 18 ºC had both PSA bristles present (Fig. 2b).

The phenotype of the absence of the posterior supraalar bristle is fully rescued in flies with the double inversion (Fig. 2). The most distal enhancer of the AS-C locus is responsible for the development of PSA bristles (Fig. 1a). The complete rescue of the phenotype in flies with the secondary inversion indicates that the phenotype of the developmental anomalies of these bristles is caused not by the break in AS-C itself, but by the effect of heterochromatin on the distal part of the locus. Additional confirmation of the predominant effect of heterochromatin on the PSA phenotype is the complete restoration of the normal PSA phenotype in flies carrying In(1) sc8 and the position effect modifier Su(var)3-906 (n = 783).

Interestingly, the proportion of males with abnormalities in the number of PSA bristles is significantly lower, at only 12 % at 25 ºC, although it rises to 42 % at 18 ºC, in accordance with the classical understanding of the enhancement of the heterochromatin position effect at lower temperatures (Elgin, Reuter, 2013). This potentially explains why PSA disruption is not documented for flies with In(1)sc8 in A. García-Bellido’s (1979) article. In that work, only hemizygous males of In(1) sc8 were analyzed, which is mentioned in a separate comment. The authors may have aimed to emphasize the weak phenotype in carriers of this inversion compared to other rearrangements affecting AS-C. A more pronounced influence of the inversion sc8 on the PSA phenotype in females was also observed in the work by D.P. Furman and V.A. Ratner (1977).

**Fig. 3.**
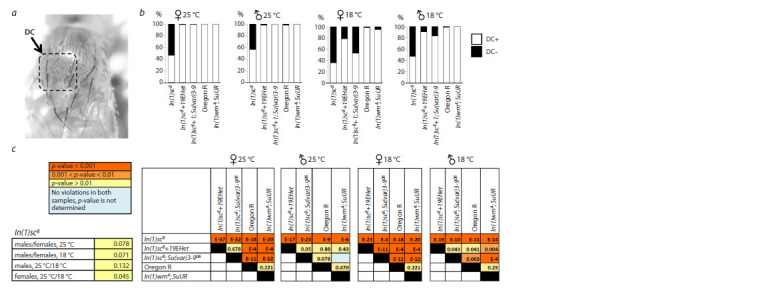
The effect of heterochromatin removing and the position effect variegation modifier Su(var)3-906 on the development of dorsocentral bristles in In(1)sc8 flies a – additional dorsocentral bristles in flies of the In(1)sc8 line; b – the proportion of flies with abnormalities in the number of dorsocentral bristles in the analyzed lines; c – pairwise comparison of the proportions of flies with altered bristle counts among different genotypes. The p-value for each comparison, calculated using the Chi-square test, is indicated

Another phenotype described in the literature for In(1)sc8 flies is the appearance of additional bristles in the dorsocentral zone (García-Bellido, 1979; Lindsley, Zimm, 1992) (Fig. 3a). We observed this phenotype in both females and males of In(1)sc8 (Fig. 3b). The additional bristles most often formed orderly rows with pairs of PDC and ADC bristles. Instances of absence of individual dorsocentral bristles were also noted. In flies of In(1)sc8+19EHet as well as In(1)sc8; Su(var)3-906, reared at 25 °C, we saw almost complete restoration of the phenotype. When culturing flies at 18 °C, the proportion of carriers with the mutant phenotype was higher in the In(1)sc8 line and was only partially, but significantly reduced against the background of the position effect modifier and the 19EHet inversion. Dorsocentral bristles develop from proneural clusters, where AS-C expression is regulated by enhancer 2, a distal enhancer that is closer to the break point of the inversion than the enhancer controlling the development of PSA. It can be assumed that the effect of heterochromatin on this enhancer is stronger. According to the data from nanopore sequencing of In(1)sc8+19EHet flies, at least 30 kb of satellite DNA 1.688 continues to flank the break point of the In(1)sc8 inversion (Kolesnikova et al., 2022).

**Fig. 4.**
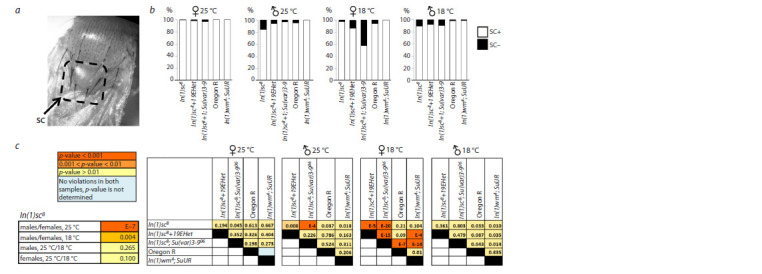
Analysis of the effect of heterochromatin removing and the position effect variegation modifier Su(var)3-906 on the development of scutellar bristles in In(1)sc8 flies a – additional scutellar bristles in the In(1)sc8 line; b – the proportion of flies with abnormalities in the number of scutellar bristles in the lines In(1)sc8, In(1) sc8+19EHet, In(1)sc8; Su(var)3-906 and two control lines, Oregon R and In(1)wm4; SuUR; c – pairwise comparison of the proportions of flies with altered bristle counts among different genotypes. The p-value was calculated using the Chi-square test.

In some literature sources, it is noted that mutants In(1)sc8 are characterized by abnormalities in the number, thickness, and length of scutellar bristles (Lindsley, Zimm, 1992; Belyaeva et al., 2003; Golovin et al., 2003). However, we did not observe a significant increase in the proportion of flies with additional or absent scutellar bristles in In(1)sc8 compared to control lines – under certain conditions, the proportion of flies with scutellar bristle abnormalities was higher in the control than in In(1)sc8 flies (Fig. 4).

**Fig. 5.**
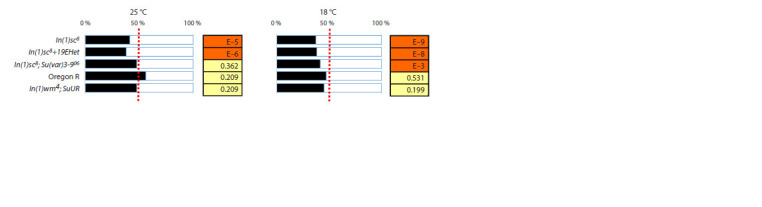
Decrease in the proportion of males in lines carrying the In(1)sc8 inversion. The ratio of females to males is shown for flies reared at temperatures of 25 and 18 °C in lines harboring In(1)sc8 and in control lines. For each comparison, the p-value calculated using the Chi-square test is indicated.

Another phenotype presumably associated with the heterochromatin effect on AS-C in flies with the In(1)sc8 inversion is the decrease in the proportion of males in the offspring. This phenotype is most pronounced in the absence of the Y chromosome, which serves as the primary evidence that it is related to a position effect (Lindsley, Zimm, 1992; Belyaeva et al., 2003). All lines carrying both In(1)sc8 and the double inversion exhibited a significant decrease in the male proportion in the offspring (Fig. 5). This ratio did not change in response to the removal of heterochromatin by the secondary inversion, nor did it depend on temperature; however, at a temperature of 25 °C, it was restored in the presence of Su(var)3-906. Thus, this phenotype is not associated with the distal part of AS-C.

Since the mutant phenotype associated with the absence of PSA bristles is observed in a large proportion of flies in the

In(1)sc8 line, and the probability of its manifestation depends on modifier factors affecting position effects variegation, such as temperature and Su(var)3-9, we decided to use In(1)sc8 as a model system to test whether a mutation in the Rif1 gene acts as a modifier of position effect. The Rap1 interacting factor 1 (Rif1) protein is an evolutionarily conserved protein that participates in various processes, including telomere length regulation, DNA repair, and establishing the temporal order of replication origin activation (Richards et al., 2022). In D. melanogaster, Rif1 is involved in establishing the late replication program of satellite sequences during embryogenesis (Sreesankar et al., 2015; Seller, O’Farrell, 2018) and is responsible for the underreplication of heterochromatin, including satellite DNA, in polytene chromosomes (Munden et al., 2018; Kolesnikova et al., 2020). To date, there are no data on whether this protein can influence the heterochromatin effect on the expression of genes positioned near blocks of satellite DNA due to chromosomal rearrangements (i. e., whether it acts as a modifier of position effect variegation).

**Fig. 6.**
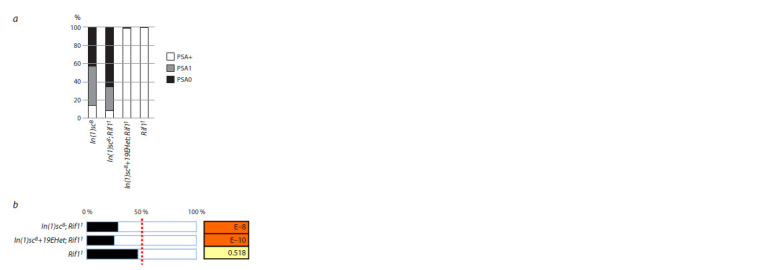
Analysis of the effect of the Rif11 mutation on the phenotypes of flies with the In(1)sc8 inversion a – the results of the comparison of the ratio of normal flies to flies with disrupted PSA bristles for females reared at 18 °C in the lines In(1)sc8, In(1) sc8; Rif11, In(1)sc8+19EHet; Rif11, and Rif11 are presented; b – the Rif11 mutation does not rescue the phenotype of decreased male proportion in flies carrying In(1)sc8.

We compared females of the lines In(1)sc8, In(1)sc8; Rif11, In(1)sc8+19EHet; Rif11, and Rif11, cultured at 18 °C (Fig. 6). Replacing chromosome 2 in the In(1)sc8 line with chromosome 2 carrying the Rif11 mutation does not restore the normal number of PSA bristles. Moreover, a slight enhancement of the phenotype is observed. A similar small enhancement of the phenotype was noted in terms of the decrease in the proportion of males. Comparing with the effect of Su(var)3-906, we can conclude that Rif11 is not a suppressor of the position effect related to the influence of satellite 1.688 on AS-C in In(1)sc8 mutants.

## Discussion

The AS-C locus is a classic model system for studying various aspects of genetic regulation of development in multicellular organisms. The vast array of easily analyzable visible phenotypes and the direct connection between individual regulatory elements and the development of specific bristles have made this locus highly attractive to researchers since the 1930s, and interest in it remains strong to this day. A deep understanding of how this locus is structured and regulated, as well as its role in the development of Drosophila, has been achieved (Modolell, Campuzano, 1998; Gómez-Skarmeta et al., 2003; Furman, Bukharina, 2019; Bukharina et al., 2023). However, some patterns and peculiarities in the behavior of mutant alleles discovered in the 1930s to 1970s have only recently become clear. A vivid example is the discovery of the hypermorphic allele of the Notch gene found in lines carrying the w a mutation (Rice et al., 2015): it turned out that all balancer X chromosomes carrying In(1)sc8 differ significantly in the expression of the sc phenotype depending on the presence of the w a allele and the linked opa33b allele of the Notch gene in the chromosome. Indirect evidence of the importance of the Notch gene status is the significant difference in the manifestation of the mutant phenotype in flies carrying the In(1)scV2 and In(1)sc8 inversions, which have closely spaced breakpoints (Rice et al., 2015): in flies of the In(1)scV2 line, the disruptions in the bristle pattern are significantly stronger, affecting more bristles. Such a strong effect of the genetic background likely complicated the interpretation of the observed patterns in the manifestation of mutations in the AS-C locus, as many sc alleles used in genetic studies were created based on the X chromosome carrying wa (Sidorov, 1931; Furman, Ratner, 1977).

The heterochromatic position of the breakpoints raises the question of the role of position effects in the manifestation of inversion phenotypes. Molecular analysis of the inversion breakpoints showed that both parts of the cluster were adjacent to large blocks of satellite 1.688 (Miller et al., 2016). Studies from the 1970s failed to reach a conclusive understanding of the role of heterochromatin (Ratner, Furman, 1978), although it was noted that in lines with the In(1)scV2 and In(1)sc8 inversions, a temperature and sex effect was observed, which differed from that in other alleles (Furman, Ratner, 1977) and supported the hypothesis of heterochromatin’s effect on the AS-C locus. In our work, these patterns were confirmed.

The position effect of heterochromatin on the phenotype of In(1)sc8, most prominently expressed in males of the X0 genotype, was described in a study by E.S. Belyaeva et al. (2003), which analyzed the effect on scutellar bristles. In our work, we did not observe a pronounced phenotype associated with scutellar bristle disruptions. It is possible that the differences are related to the criteria for disruption adopted for the analysis: we considered as a disruption only the excess or absence of bristles, while the work of E.S. Belyaeva et al. mentioned changes in their thickness and length. By applying stricter criteria to identify disruptions, we did not find abnormalities in bristle number, for which, according to the literature, proximal enhancers of AS-C are required. The work of A.K. Golovin et al. (2003) shows that the phenotype of flies with mutations In(1)scV2 and In(1)sc8 is significantly enhanced by mutations in the genes su(Hw) or mod(mdg4), and this enhancement affects the scutellar bristles. The authors conclude that under normal circumstances, the effect of heterochromatin on the proximal part of AS-C in In(1)scV2 and In(1)sc8 mutants is blocked by an unannotated insulator. The presence of a wellstudied insulator localized between the regulatory region of AS-C and the yellow gene (Golovin et al., 2003) explains the weak effect of heterochromatin on the gene yellow – the effect is observed only in X0 males (Lindsley and Zimm, 1992; Belyaeva et al., 2003).

Two sc alleles – In(1)scV2 and In(1)sc8 – are associated with inversions, one of the breakpoints of which is located within the AS-C locus between the ac and sc genes, while the second is in the pericentric heterochromatin (Miller et al., 2016). Elegant studies analyzing the expression of the ac and sc genes in In(1)sc8 mutants showed that the locus, split into two parts, can continue to function normally because the functions of these genes largely duplicate each other. Under normal conditions, both proximal and distal enhancers influence the expression of each of the ac and sc genes, resulting in both proteins being detected in all proneural clusters during immunostaining of imaginal discs with antibodies to the Ac and Sc proteins. In carriers of In(1)sc8, the corresponding regulatory element in each proneural cluster of the imaginal disc activates the expression of only one of the genes (either ac or sc), but this is sufficient to form a nearly normal bristle pattern (Gómez-Skarmeta et al., 1995). Moreover, this is sufficient for the complete restoration of the phenotype when the block of heterochromatin is removed from the distal part of the cluster or when there is a strong modifier of the position effect variegation. We obtained direct evidence that, with a reduction in the effect of heterochromatin, the rearranged AS-C locus can provide a normal phenotype in flies. This observation is interesting from the perspective of the evolution of loci with complex regulatory systems: the protein-coding genes that arose as a result of duplication with redundant functions may, due to chromosomal rearrangements, divide their functions and start evolving along independent trajectories. It is known that the ac and sc genes are the result of relatively recent duplication; outside of the Drosophila group, the homologs of ac and sc are represented by a single gene (Negre, Simpson, 2009).

Using the contrasting effect of heterochromatin in flies carrying In(1)sc8 on the PSA phenotype, we decided to test whether the Rif1 protein can modify this effect. In D. melanogaster, the Rif1 protein is involved in establishing the late replication program of satellite sequences during embryogenesis (Seller, O’Farrell, 2018). In the polytene chromosomes, mutations in the Rif1 gene completely suppress underreplication in heterochromatic regions, including the replication of satellite DNA (Kolesnikova et al., 2020).

In cells with polytene chromosomes, Rif1 interacts with the suppressor of underreplication (SuUR) protein (Nordman et al., 2018), which is a weak modifier of the position effect in D. melanogaster (Belyaeva et al., 2003). Therefore, one would expect that in Rif11 mutants, the properties of heterochromatin could significantly differ from the norm; particularly, the effect of heterochromatin on the transcription of adjacent genes in chromosomal rearrangements could change. We did not observe a suppressive effect of the Rif11 mutation on the position effect associated with the satellite 1.688 effect on the distal part of the AS-C cluster in the In(1)sc8 inversion, nor on the sex ratio in the offspring of flies carrying this inversion. Moreover, we detected a weak enhancer effect, the evidence of which requires further verification

## Conclusion

In summary, we can conclude that the phenotype associated with the disruption of the bristle pattern in In(1)sc8 mutants is primarily caused not by the splitting of the AS-C locus into two parts, but by the effect of heterochromatin on the distal part of the cluster. This can be used to test the influence of various factors on heterochromatin-induced position effect variegation

## Conflict of interest

The authors declare no conflict of interest.
